# Incidence of Human *Taenia solium* Larval Infections in an Ecuadorian Endemic Area: Implications for Disease Burden Assessment and Control

**DOI:** 10.1371/journal.pntd.0002887

**Published:** 2014-05-22

**Authors:** Marco Coral-Almeida, Richar Rodríguez-Hidalgo, Maritza Celi-Erazo, Héctor Hugo García, Silvia Rodríguez, Brecht Devleesschauwer, Washington Benítez-Ortiz, Pierre Dorny, Nicolas Praet

**Affiliations:** 1 Institute of Tropical Medicine, Department of Biomedical Sciences, Antwerp, Belgium; 2 Universidad Central del Ecuador, Centro Internacional de Zoonosis (CIZ), Ciudadela Universitaria, Quito, Ecuador; 3 Ghent University, Faculty of Veterinary Medicine, Merelbeke, Belgium; 4 Universidad Central del Ecuador, Facultad de Medicina Veterinaria y Zootecnia, Ciudadela Universitaria, Quito, Ecuador; 5 Universidad Central del Ecuador, Núcleo de Investigadores, Ciudadela Universitaria, Quito, Ecuador; 6 Universidad Peruana Cayetano Heredia, Lima, Perú; 7 Instituto Nacional de Ciencias Neurológicas, Lima, Perú; 8 Université catholique de Louvain, Institute of Health and Society, Brussels, Belgium; Universidad Nacional Autónoma de México, Mexico

## Abstract

**Background:**

Human cysticercosis is a zoonotic disease causing severe health disorders and even death. While prevalence data become available worldwide, incidence rate and cumulative incidence figures are lacking, which limits the understanding of the *Taenia solium* epidemiology.

**Methodology/Principal findings:**

A seroepidemiological cohort study was conducted in a south-Ecuadorian community to estimate the incidence rate of infection with and the incidence rate of exposure to *T. solium* based on antigen and antibody detections, respectively. The incidence rate of infection was 333.6 per 100,000 person-years (95% CI: [8.4–1,858] per 100,000 person-years) contrasting with a higher incidence rate of exposure 13,370 per 100,000 person-years (95% CI: [8,730–19,591] per 100,000 person-years). The proportion of infected individuals remained low and stable during the whole study year while more than 25% of the population showed at least one antibody seroconversion/seroreversion during the same time period.

**Conclusions/Significance:**

Understanding the transmission of *T. solium* is essential to develop ad hoc cost-effective prevention and control programs. The estimates generated here may now be incorporated in epidemiological models to simulate the temporal transmission of the parasite and the effects of control interventions on its life cycle. These estimates are also of high importance to assess the disease burden since incidence data are needed to make regional and global projections of morbidity and mortality related to cysticercosis.

## Author Summary

Human cysticercosis is a neglected zoonotic parasitic disease causing severe health disorders such as epilepsy and even death. Cysticercosis is related to poverty, inadequate hygiene conditions and traditional pig farming. The present study describes the dynamic nature of human *Taenia solium* larval infections in an Ecuadorian endemic community. In this study we report for the first time incidence rate and cumulative incidence figures of human *T. solium* larval infections in Latin America. The simultaneous use of antibody and antigen serological detections allowed estimating both parasite exposure and infection rates, respectively. While about 13% of the inhabitants were exposed to *T. solium* eggs, less than 1% of the population became yearly infected with the parasite. This contrast between exposure and infection may be linked to an effective resistance to the parasite acquired through long-term exposure of the population and differs from the African situation, where much higher levels of infection have been observed. These estimates are of high importance to understand the epidemiology of *T. solium* in order to develop ad hoc cost-effective prevention and control programs. They are also essential to assess the burden of *T. solium* cysticercosis since longitudinal data are needed to make regional and global projections of morbidity and mortality related to cysticercosis.

## Introduction

Human cysticercosis (CC) is a parasitic disease caused by the development of the metacestode larval stage of *Taenia solium* (cysticercus) in the muscles, the central nervous system (causing neurocysticercosis (NCC)), the subcutaneous tissue and the eyes (causing subcutaneous and ocular cysticercosis, respectively) [1]. The life cycle of the parasite includes humans as sole definitive hosts and pigs as main intermediate hosts. Humans get infected by consumption of raw or undercooked pork infected with cysticerci, resulting in the development of an adult intestinal tapeworm (taeniosis). Pigs become infected by ingestion of *T. solium* eggs contained in infected human feces, through coprophagic behavior or via ingestion of contaminated water or food, and develop porcine CC. Man can also act as a dead-end intermediate host by accidental ingestion of *T. solium* eggs [2] and develop human CC. NCC may cause severe neurological disorders and even death [3], [4]. It is the most important parasitic disease of the central nervous system and the main cause of acquired epilepsy in *T. solium* endemic areas, where NCC is associated with 14.2 to 50% of the epilepsy cases [5], [6]. The maintenance of the parasite life cycle is associated with poor sanitation, lack of hygiene and traditional pig rearing systems allowing free roaming of the animals. Endemic areas have been identified in Asia, Africa and Latin America [7]–[10]. In Latin America the infection has been reported in at least 18 countries and is considered a major public health problem, especially in poor rural areas [7], [8]. The Andean region of Ecuador and neighboring countries is hyper-endemic for cysticercosis [11]. While reliable prevalence data become available worldwide, they may considerably vary depending on the diagnostic test used [12]–[14]. Several tools are available for the diagnosis of human CC, i.e. imaging and serological techniques. Serological antigen and antibody detections are valuable tools when conducting epidemiological studies, since they inform on infection with and exposure to the parasite, respectively. Taking the latter distinction into account, studies conducted in Ecuadorian endemic rural communities have shown an exposure to the parasite ranging from 25 to 40% and a proportion of infected individuals ranging from 2.25 to 4.99% [15]–[17]. However, prevalence figures do not inform on the evolution of the number of positive cases over time and estimates for human cysticercosis incidence rate and cumulative incidence are lacking, which limits the understanding of the transmission dynamics of *T. solium* and does not allow a precise estimation of its disease burden. García *et al.* (2001) [18] conducted longitudinal studies in endemic areas of Peru and Colombia and demonstrated the presence of transient antibody responses suggesting a high number of antibody seroconverted cases per year ranging from 8 to 25% of the population depending on the studied area. Through rule-based modeling, Praet *et al.* (2010) [16] simulated the annual antibody seroconversion rate in an endemic area of Ecuador. They estimated an annual incidence rate of exposure of people becoming seropositive of 14 per 100 person-years. On the other hand, studies estimating both incidence rate of infection and cumulative incidence are scarce [16], [18], [19]. Mwape et al. (2013) [20] reported an incidence rate of infection of 6,300 per 100,000 person-years in a rural community of eastern Zambia. Such estimates for Latin America are inexistent. For this reason, the present study aims at estimating the cumulative incidence and the incidence rate of human CC in an endemic area of Ecuador. A sero-epidemiological cohort study was conducted to investigate the transmission dynamics of *T. solium* among individuals living in a southern Ecuadorian rural community. This paper reports estimates of the incidence rates, cumulative incidences of active infection and exposure rates to *T. solium* and discusses the implications for the disease burden assessment and control.

## Materials and Methods

### Ethical clearance

The protocol used in this study was approved by the Ethical Committee of the Central University of Ecuador (IRB 00002438) and by the Ethical Committee of The University Hospital of Antwerp, Belgium. Written informed consent was obtained from each individual willing to participate in the study. For participants aged less than 18 years old written informed consent was also obtained from a parent or a legal adult representative. Individuals testing positive for *T. solium* cysticercosis antigens were referred to the local health center for follow-up.

### Study area, population and design

The study was conducted in the rural parish of Sabanilla (4° 12′S, 80° 8′W) belonging to the Celica canton in the Southern Ecuadorian province of Loja. The parish has 1145 inhabitants; most of them are farmers involved in activities related to agriculture and animal husbandry. The climate is semi-arid, and the altitude is 700 meters above sea level. The region is endemic for *T. solium* cysticercosis and presents the risk factors for the transmission of the parasite [15], [21]. A sero-epidemiological community-based cohort study was performed. Three blood sampling rounds were organized in Sabanilla in a period of 13 months: the first sampling round took place in June 2009 (SR1), the second in November 2009 (SR2) and the third one in July 2010 (SR3). Based on the three sampling rounds, three periods of time were defined as follows: a six-month period from June 2009 to November 2009 (P1), a seven-month period from November 2009 to July 2010 (P2), and a total 13-month period from June 2009 to July 2010 (P3).

First, an informative meeting inviting the population to participate took place at the beginning of the study in collaboration with the local authorities. Then, a census of the population was conducted based on a door-to-door survey, including collection of information on age and sex of the inhabitants. After informed consent, all individuals older than one year willing to participate and present at the time were blood sampled.

### Samples collection and analyses

At each sampling round, 10 ml of blood was collected in dry tubes. After coagulation and centrifugation, serum was collected and stored at −20°C until analysis. Two serological diagnostic tests were performed. (1) The Enzyme Linked Immunosorbent Assay for the detection of circulating antigens of the metacestode of *T. solium* (Ag-ELISA) [22]–[24]. The sensitivity and specificity of the Ag-ELISA for detecting active infection in humans are 90% (95% CI: [80–99%]) and 98% (95% CI: [97–99%]), respectively. No cross-reaction with other parasites has been reported [13], [23]. (2) The Enzyme-Linked Immunoelectrotransfer Blot (EITB) for the detection of antibodies directed against seven specific *T. solium* metacestode glycoproteins [25]. The sensitivity and specificity of the EITB for detecting exposure to the parasite range from 97% to 98% and from 97% to 100%, respectively [13], [25].

### Antigen and antibody seroprevalence

The antigen and antibody seroprevalence (Ag and Ab seroprevalence), as based on the results of the Ag-ELISA and of the EITB, respectively, were calculated for each sampling round for the whole population and by sex.

A multinomial Bayesian model adapted from Berkvens et al. (2006) [26] was used to estimate the true prevalence of *T. solium* larval infections for each sampling round based on the antigen seroprevalence data and on prior information on the test characteristics (sensitivity and specificity of the Ag-ELISA). Prior information was extracted from the available literature [13]. A uniform distribution with lower and upper limits of 0.80 and 1.00, and 0.97 and 1.00 were used to constrain the sensitivity and the specificity of the test, respectively. The analysis was conducted in WinBUGS and R [27], [28]. Three chains, 20,000 iterations, following a burn-in of 5,000 were used to assess the convergence of the results. Criteria assessing the fit between prior information and the seroprevalence data were evaluated, i.e. the Bayesian p-value (Bayesp), the Deviance Information Criterion (DIC) and the number of parameter effectively estimated by the model (pD) [26], [28].

### Seroconversion, seroreversion and incidence rate

First, proportion of change to antigen seropositivity/seronegativity (change to Ag seropositivity/seronegativity) and proportion of antibody seroconversion and seroreversion (Ab seroconversion and seroreversion) were calculated to characterize the transmission dynamics of the disease.

Seroconversion is defined as the change from a negative to a positive serological test result between 2 sampling rounds; the opposite is defined as seroreversion [29]. The proportion of Ab seroconversion and the proportion of change to Ag seropositivity reflect the cumulative incidence for a defined time period. They were calculated by dividing the number of new cases by the number of susceptible individuals (having a negative test result at the previous sampling round) during a given time. The proportion of Ab seroreversion and the proportion of change to Ag seronegativity were calculated by dividing the number of positive tests that turned negative by the number of positive tests at the previous sampling round.

The incidence rate of infection with the larval stage of *T. solium* and the incidence rate of exposure to *T. solium* eggs were also calculated based on the results of the antigen and antibody detection tests, respectively.

The incidence rate was calculated as the number of new (change from seronegativity to seropositivity) cases in a defined time period divided by the number of person-time units at risk during the time-period. Yearly incidence rates were multiplied by 100,000 to be expressed by 100,000 person-years [30], [31]. The person-time unit represents one person for a defined period of time. The latter was calculated as described in Ngowi et al. (2008) assuming that the infection occurs uniformly over time and considering halfway the period between two sampling rounds [32]. For example, if a person is followed up for six months and does not seroconvert during this time, this person will contribute 0.5 person-year to the person-time at risk. If a person that is followed up for the same period but seroconverts during that period, this person will contribute 0.25 person-year to the person-time at risk. Yearly incidence rates were calculated based on this calculation method. Ninety-five % exact Poisson confidence intervals were calculated using the epitools package in R for all incidence rates [33].

### Data management and statistical analyses

Data were entered in Excel 2010 (Microsoft Office 2010). Statistical analyses were performed in Stata (Stata Corp., College Station, TX) and in R: [34]

Fisher exact test was used to compare (1) Ag/Ab seroprevalence between sex within each sampling round and (2) Ag seroprevalence with Ab seroprevalence within each sampling rounds. Also, McNemar test was performed to compare the sero-Ag and sero-Ab prevalence between rounds. Multivariate logistic regression analysis was used to study the association between sero-Ag/Ab prevalence and age and sex, and this for the three samplings rounds. The significance level was set at 0.05.Fisher exact test was used to compare (1) the proportion of Ab seroconversion with the proportion of antibody seroreversion and the proportion of change to Ag seropositivity with the proportion of change to Ag seronegativity within periods, (2) the proportions of Ab seroconversion/seroreversion and the proportions of Ag change to seropositivity/seronegativity between sexes, (3) the proportions of Ab seroconversion/seroreversion and the proportions of change to Ag seropositivity/seronegativity between periods.

In addition, a change point analysis was used to compare the proportion of Ab seroconversion with the proportion of Ab seroreversion in function of age. The change point analysis classifies the population into 2 age groups at different age points (10, 20, 30, 40, 50, 60, 70, 80 years old). The Fisher exact test was then used on both age groups in order to identify any change of significance when comparing the proportions of Ab seroconversion and seroreversion [16], [20], [35]. The significance level was set at 0.05 for all statistical analyses.

## Results

**Figure 1:**
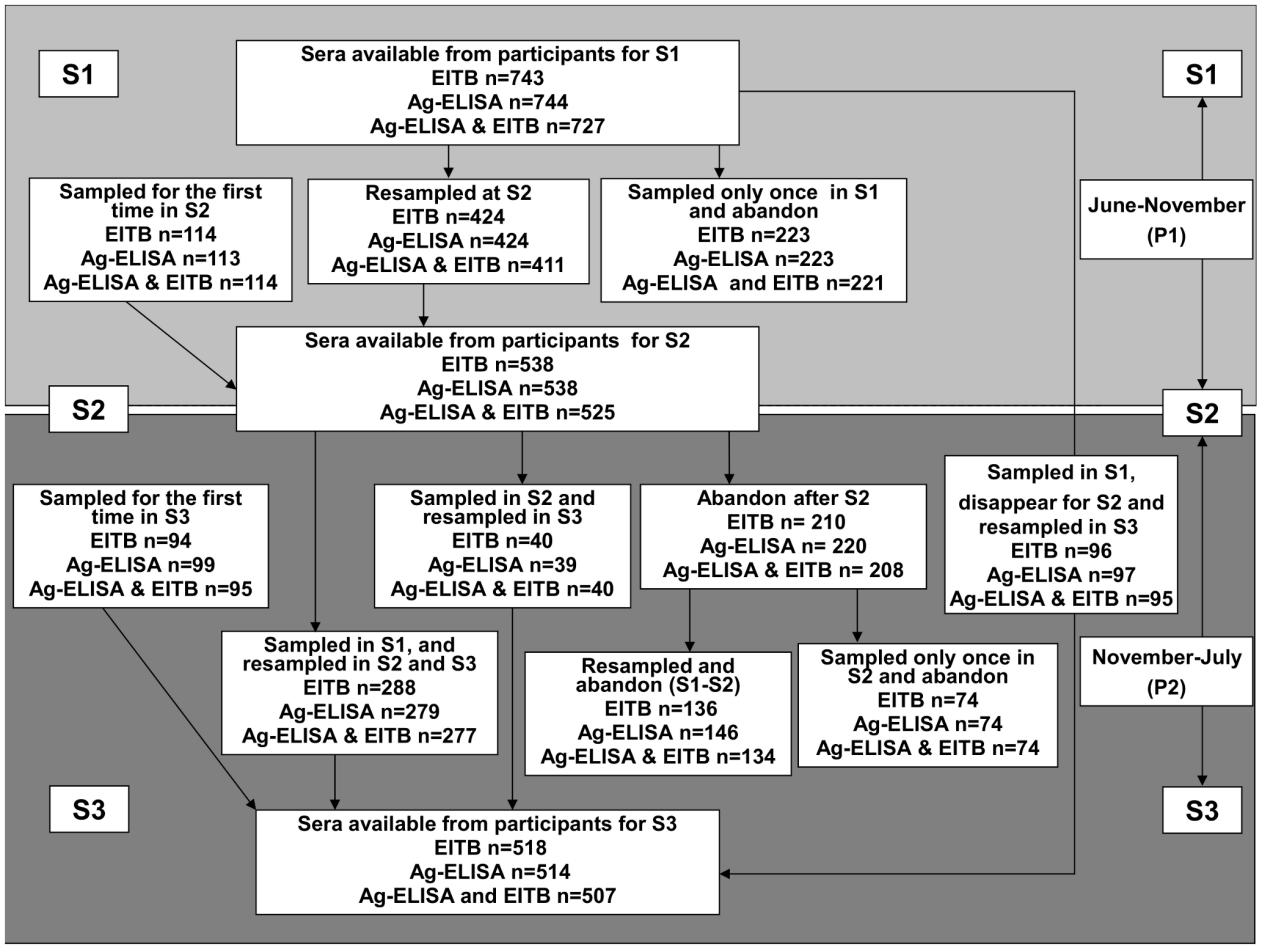
Sera availability and individual participation during the three sampling rounds. (Format adapted from Mwape et al., 2013). S1, S2 and S3 stand for first, second and third sampling rounds, Ag-ELISA: Enzyme Linked ImmunosSorbent Assay for the detection of circulating antigens of the metacestode of T. solium, EITB: Enzyme-Linked Immunoelectrotransfer Blot for the detection of antibodies directed against seven specific T. solium metacestode glycoproteins.

A total of 967 (84.45%) individuals from the 1145 inhabitants listed in the census participated in the study. Depending on the willingness of the individuals to participate, their presence at the time of sampling and on the quantity of serum available, EITB was performed on 743 (64.9%), 538 (47%) and 518 (45.2%) sera for June, November and July, respectively. Ag-ELISA was performed on 744 (65%), 538 (47%) and 514 (44.9%) sera for the same time periods. Figure 1 describes in detail sera availability and individual participation during the three sampling rounds.

### Antigen and antibody seroprevalence

The Ag and Ab seroprevalence for each sampling round for the whole population and by sex are presented in Table 1. The prevalence adjusted for misclassification error of *T. solium* larval infections was 0.7% (95% Credibility Interval (CI): [0.03–1.75]), 0.7% (95% CI: [0.03–2.00]) and 1.1% (95% CI: [0.05–2.84]) for SR1, SR2 and SR3, respectively. All except one Ag positive individuals were also Ab positive in the 3 sampling rounds. Fisher exact test did not reveal any significant difference of Ag and Ab seroprevalence between sexes. McNemar test did not reveal any significant difference of Ag and Ab seroprevalence between rounds. Ab seroprevalence was significantly higher than Ag seroprevalence within each sampling round. Multivariate logistic regression analysis showed a significant positive correlation between Ab seroprevalence and age.

**Table 1:** Antigen and antibody seroprevalence figures (as based on the results of the Ag-ELISA and the EITB, respectively) by sex and by sampling round.

Seroprevalence	Sampling round	Sex	Number of positive cases/Total number of individuals	Percentage of positive cases [95%CI]^a^
**Antigen**	SR 1^b^	Female	2/367	0.5 [0.1–2]
		Male	5/377	1.3 [0.4–3.1]
		Total	7/744	0.9 [0.4–1.9]
	SR 2^c^	Female	1/293	0.3 [0–1.9]
		Male	4/245	1.6 [0.4–4.1]
		Total	5/538	0.9 [0.3–2.2]
	SR 3^d^	Female	3/265	1.1 [0.2–3.3]
		Male	5/249	2 [0.7–4.6]
		Total	8/518	1.5 [0.7–3]
**Antibody**	SR 1^b^	Female	124/364	34.1 [29.2–39.2]
		Male	108/379	28.5 [24–33.3]
		Total	232/743	31.2 [27.9–34.7]
	SR 2^c^	Female	100/293	34.1 [28.7–39.9]
		Male	83/245	33.9 [28–40.2]
		Total	183/538	34 [30–38.2]
	SR 3^d^	Female	80/270	29.6 [24.2–35.5]
		Male	77/248	31 [25.3–37.2]
		Total	157/518	30.3 [26.4–34.5]

a CI = Binomial exact 95% Confidence Intervals; SR = sampling round;

b SR 1 = June 2009 sampling,

c SR 2 = November 2009 Sampling;

d SR 3 = July 2010 Sampling.

Table 2 shows the proportions of antigen and antibody seropositive and/or seronegative individuals who participated in all 3 sampling rounds and whose sera were available for both tests (n = 277). Only one individual changed to antigen seropositivity status throughout the entire study period. Eighteen percent of this restricted population remained antibody positive throughout the entire study period while about 20% of the individuals showed at least 1 change of antibody positivity status.

**Table 2:** Proportions of antigen and antibody positive and/or negative individuals who participated in the 3 sampling rounds.

Test result per sampling round			Number of individuals (antibody detection)	Percentage of individuals (antibody detection; [95%CI]^a^)	Number of individuals (antigen detection)	Percentage of individuals (antigen detection; [95%CI]^a^)
SR 1^b^	SR 2^c^	SR 3^d^				
−	−	−	169	61 [54.99–66.7]	276	99.63 [98–99.99]
−	−	+	9	3.24 [1.49–0.6]	1	0.36 [0.01–1.99]
−	+	−	8	2.89 [1.25–5.61]	0	…
−	+	+	9	3.24 [1.49–6.07]	0	…
+	−	−	13	4.69 [2.52–7.89]	0	…
+	−	+	4	1.44 [0.36–3.66]	0	…
+	+	−	14	5.05 [2.79–8.33]	0	…
+	+	+	51	18.4 [14.02–23.49]	0	…
**Total**			277		277	

a CI = Binomial exact 95% Confidence Intervals; SR = sampling round;

b SR 1 = June 2009 sampling,

c SR 2 = November 2009 Sampling;

d SR 3 = July 2010 Sampling.

### Seroconversion, seroreversion and incidence rate

The overall incidence rate of human *T. solium* larval infection based on -antigen detection was 333.6 per 100,000 person-years (95% exact Poisson CI: [8.4–1,858] per 100,000 person-years). The overall incidence rate of exposure to *T. solium* based on antibody detection was 13,370 per 100,000 person-years (95% exact Poisson CI: [8,730–19,591] per 100,000 person-years). Ag proportion of changes to seropositivity/seronegativity and proportion of Ab seroconversion/seroreversion by period are represented in Table 3. Incidence rates estimates for individuals who participated in at least two of the sampling rounds are given in Table 4.

**Table 3:** Antigen change to seropositivity/seronegativity test result, antibody seroconversions/seroreversion figures (as based on the results of Ag-ELISA and EITB respectively) by period.

Test	Period	Parameter	Number of individuals	Percentage of individuals ([95%CI]^a^)
**Antigen**	P1^c^	Change to seropositivity for 6 months	0/421	0 [0–0.9]^b^
		Change to seronegativity for 6 months	0/3	0 [0–70.8]^b^
	P2^d^	Change to seropositivity for 7 months	1/317	0.3 [0–1.7]
		Change to seronegativity for 7 months	0/1	0 [0–97.5]^b^
	P3^e^	Change to seropositivity for 13 months	2/373	0.5 [0.1–1.9]
		Change to seronegativity for 13 months	1/1	1 [2.5–100]^b^
**Antibody**	P1^c^	Seroconversions	26/288	9 [6–12.9]
		Seroreversions	25/135	18.5 [12.4–26.1]
	P2^d^	Seroconversions	17/226	7.5 [4.4–11.8]
		Seroreversions	26/101	25.7 [17.6–35.4]
	P3^e^	Seroconversions	24/264	9.1 [5.9–13.2]
		Seroreversions	34/120	28.3 [20.5–37.3]

a CI = Binomial exact 95% Confidence Intervals;

b CI = one-sided, 97.5% confidence interval;

c P1 = June–November period;

d P2 = November–July period;

e P3 = June–July period.

**Table 4:** Yearly incidence rates for infection and exposure (as based on the results of Ag-ELISA and EITB, respectively) of individuals who participated in two sampling rounds by period.

Test	Period between samplings	Number of individuals	Yearly incidence rate ([95% Poisson CI]^a^) per 100,000 person-years
**Antigen**	P1^b^ (6 months)	0/421	0[0–1790.7]
	P2^c^ (7 months)	1/317	541.6[13.7–3018]
	P3^d^(13 months)	2/373	496.3 [60.1–1792.7]
**Antibody**	P1^b^ (6 months)	26/288	18,909.1[12,352–27,706.2]
	P2^c^ (7 months)	17/226	13,399[7,805.4–21,453.1]
	P3^d^ (13 months)	24/264	8,791.2[5,632.7–13,080.6]

a CI = Poisson exact 95% Confidence Intervals;

b P1 = June–November period;

c P2 = November–July period;

d P3 = June–July period.

**Figure 2:**
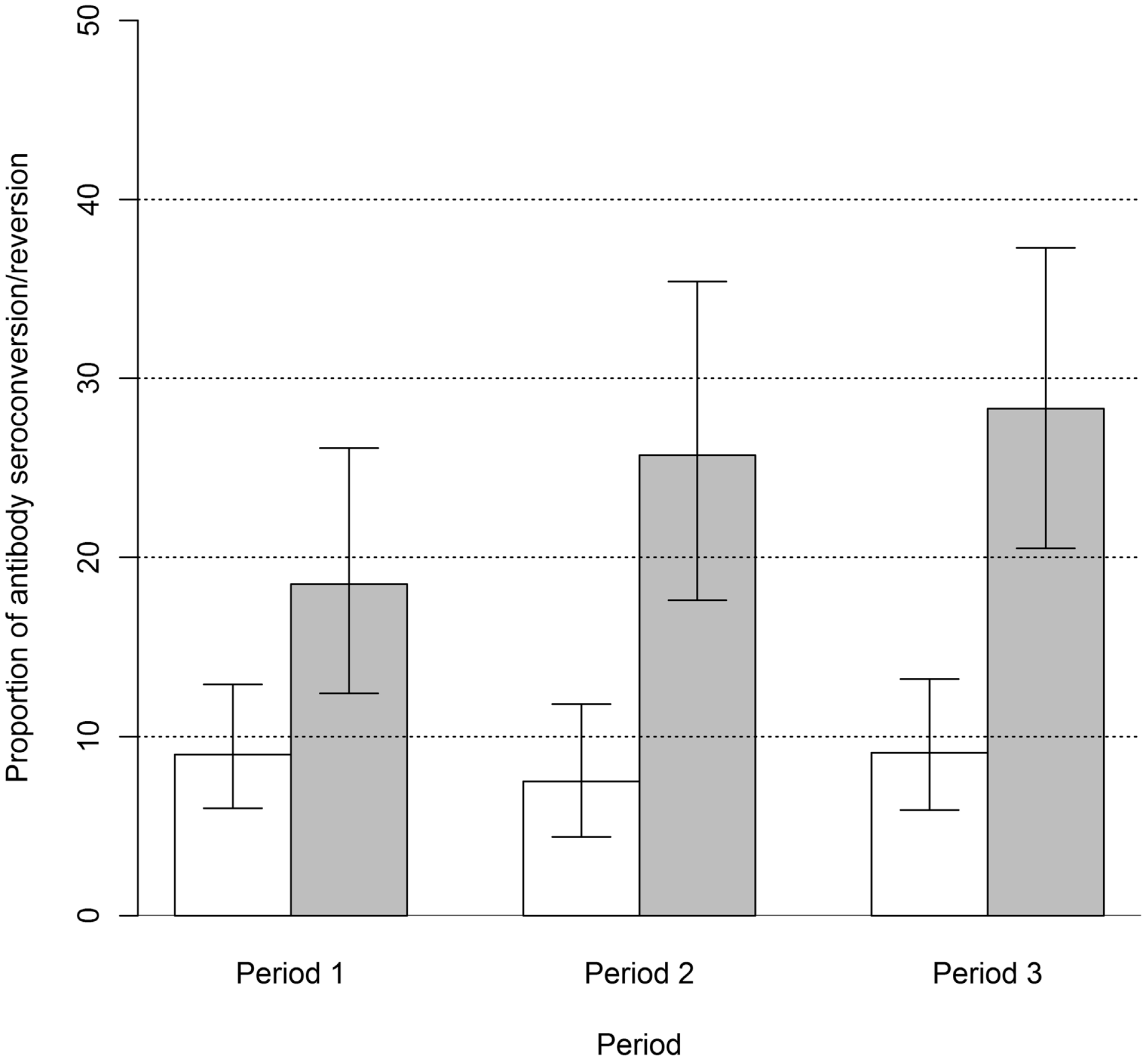
Proportions of Ab seroconversion and seroreversion by time period. White bars represent proportions of Ab seroconversion whereas grey bars represent proportions of Ab seroreversion; vertical error bars represent the upper and lower limits for the 95% binomial exact confidence interval; Period 1 stands for the period between Sampling round 1 and sampling round 2 (June–November 2009); Period 2 stands for the period between sampling round 2 and sampling round 3 (November 2009–July 2010) and Period 3 stands for the period between sampling round 1 and sampling round 3 (June 2009–July 2010).

**Figure 3:**
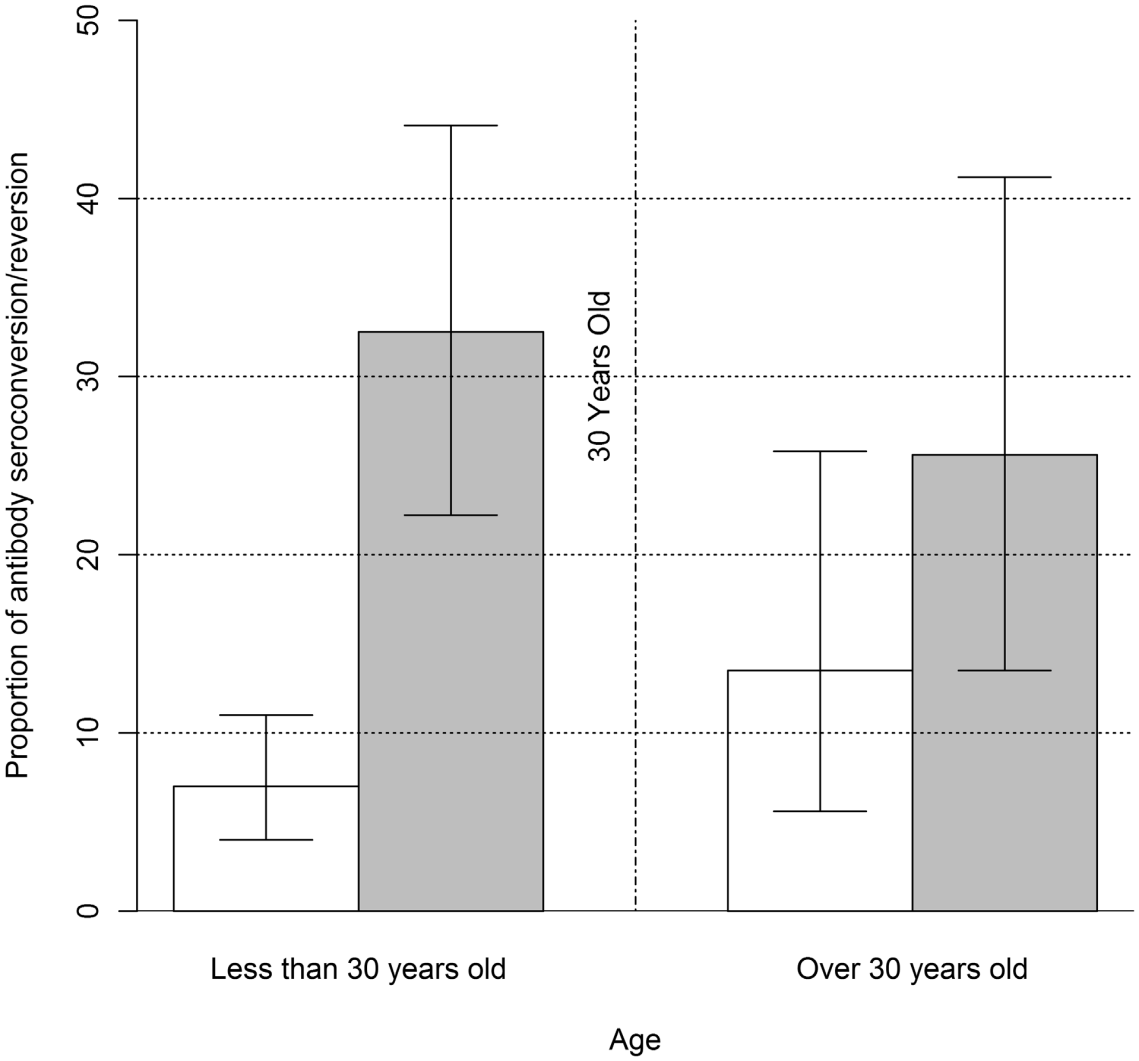
Results of the change point analysis with a change point at 30 years old. The vertical dotted/lined line represents the change point at 30 years old; white bars represent proportions of Ab seroconversion whereas grey bars represent proportions of Ab seroreversion; vertical error bars represent the upper and lower limits for the 95% binomial exact confidence interval.

Fisher exact test did not show any difference of proportion of Ab seroconversion/seroreversion and proportions of change to Ag seropositivity/seronegativity between sexes, nor between periods. Proportion of Ab seroreversion was significantly higher than proportion of Ab seroconversion for each period (Figure 2). The change point analysis showed that the proportion of Ab seroreversion was significantly higher than proportion of Ab seroconversion until the age of 30 years. After this change point, the difference was not significant (Figure 3).

## Discussion

This is the first study reporting cumulative incidence and incidence rate figures of human *T. solium* larval infection in Latin America. The overall incidence rate of infection in the endemic rural community of Sabanilla, was 333.6 per 100,000 person-years (95% exact Poisson CI: [8.4–1,858] per 100,000 person-years), which suggests that less than 1% of the population becomes infected yearly with the parasite. In contrast, the incidence rate of exposure to *T. solium* is much higher: about 14% of the population has a yearly contact with the parasite. The latter estimates are in line with observed and simulated antibody seroconversion rates ranging from 8 to 25% in Peru, Colombia and Ecuador [16], [18]. Proportions of change to Ag seropositivity/seronegativity and Ab seroconversion and seroreversion were identical in males and females indicating that both genders get equally infected with/are equally exposed to the parasite. Moreover, these proportions did not significantly vary in time (one year period). On the other hand, proportion of Ab seroreversion was significantly higher than proportion of seroconversion Ab for each period and a change point analysis showed that proportion of Ab seroreversion was significantly higher than proportion of Ab seroconversion until the age of 30 years. After this change point, the difference was not significant. These results corroborate the findings of Praet et al. (2010) [16] suggesting a higher proportion of seroreversion before the age of 40 years due to a higher number of primary immune responses before this age. In other words, individuals will serorevert more rapidly before the age of 30–40 years because primary humoral response is shorter and weaker than secondary response. Thus, the proportion of seroreversions depends on the immunological status of the individuals.

The dynamics of infection and exposure in the population, represented by the proportions of antigen and antibody results from the individuals who participated in all 3 sampling rounds, showed that the proportion of infected individuals remains low and stable during the whole study year, while the proportion of exposed individuals is remarkably higher. Of note is the high level of serological status variation with more than 20% of the population showing at least one antibody seroconversion/seroreversion during the year. Together with the prevalence estimates presented by period, these longitudinal data corroborates the findings of other studies conducted in Latin America highlighting a high prevalence of exposure to the parasite but a low prevalence of active infections. This contrast between exposure and infection may be linked to an effective resistance to the parasite acquired through long-term exposure of the population. In addition, these results confirm the occurrence of transient antibody responses in individuals living in *T. solium* endemic areas and suggest exposure to the parasite without infection or mild infections that are aborted by the natural immunity of the individual [20]. Mwape et al. (2013) [20] conducted a similar community-based longitudinal study in the Eastern Province of Zambia. While a much higher incidence rate was observed in the African endemic area, similar higher proportion of Ab seroreversion than Ab seroconversion and the presence of transient antibody responses were described. Further studies are needed to unravel the difference of parasite transmission patterns in different epidemiological settings. Specifically, research should focus on identifying the causes for differences in infection levels. In this context, the identification of tapeworm carriers in a community should be based on improved methods, because sensitivity and specificity of conventional coprological methods are low [36].

Our study has limitations that are mainly due to the inhabitant proportion of participation. Although 967 individuals from a total of 1045 inhabitants provided at least one blood sample, 396 provided only one sample and another 283 provided two blood samples. Compliance in participating in all 3 sampling rounds was of 288 volunteers despite extensive information sessions prior to the sampling procedure. The main reason for irregular participation was the absence of the individuals for professional duties. Reduced participation can have an impact on the precision of the estimation of the incidence rate, however, the participation on the three sampling rounds is still representative of the total population and all the participants selected for the incidence rate estimation match all the selection criteria for this calculation (n = 288 (27.6% (95% CI: [24.9–30.4]))). Another limitation of the study is the limited number of samplings and the relatively long sampling intervals depending on logistic, economic and ethical constraints. In other words, much more information could have been produced if more sampling had been organized at shorter time intervals, i.e. the positivity status of the participants would have been more accurately monitored over time. The incidence rate estimation for both infection and exposure is likely to be lower with increasing intervals between samplings: a proportion of the new infections may be undetected and the time of occurrence of a new infection overestimated. A quicker detection of new infections will result in a decrease of the number of person-years at risk and consequently in higher estimates of the incidence rate. Finally, even though the tests used in this study have shown high sensitivity and specificity, false positive and negative individuals may bias the prevalence and the incidence rate estimates. Bayesian estimation of infection with *T. solium* larva prevalence has been used to estimate the true prevalence of infection with an exposure to *T. solium*. The Bayesian estimation corrects the apparent prevalence at, but does not allow to know the true infection status at the individual level. Consequently, it does not allow to estimating the true incidence rate.

In conclusion, the present study underlines the importance of conducting longitudinal serological follow-up allowing generating incidence rather than prevalence data to fully understand the transmission dynamics of the infection and to avoid under/overestimation of the occurrence of the parasite. Similar cohort studies assessing the effect of risk factors such as development of immunity and behavioral factors should be conducted to identify all the parameters that may influence parasite transmission. Understanding the transmission dynamics of *T. solium* is essential to develop ad hoc cost-effective prevention and control programs. The estimates generated here may now be incorporated in epidemiological models to simulate the temporal transmission of the parasite and the effects of control interventions on its life cycle [19]. These estimates are also of high importance to assess the burden of *T. solium* cysticercosis since incidence data are needed to make regional and global projections of morbidity and mortality related to cysticercosis. To this end, the link between the incidence rate of infection and health outcomes related to human cysticercosis, such as epilepsy and chronic headache, as well as the case-fatality ratio still need to be estimated.

## Supporting Information

Checklist S1 STROBE checklist.
(DOC)
